# Testosterone and soluble ST2 as mortality predictive biomarkers in male patients with sepsis-induced cardiomyopathy

**DOI:** 10.3389/fmed.2023.1278879

**Published:** 2024-01-08

**Authors:** Lu Wang, Wen Dai, Ruiyao Zhu, Tingting Long, Zhaocai Zhang, Zhenju Song, Sucheng Mu, Shasha Wang, Huijuan Wang, Jiaxi Lei, Jing Zhang, Wenfang Xia, Guang Li, Wenwei Gao, Handong Zou, Yan Li, Liying Zhan

**Affiliations:** ^1^Department of Critical Care Medicine, Renmin Hospital of Wuhan University, Wuhan, China; ^2^Department of Clinical Laboratory, Renmin Hospital of Wuhan University, Wuhan, China; ^3^Department of Infection Prevention and Control, Renmin Hospital of Wuhan University, Wuhan, China; ^4^Department of Critical Care Medicine, The Second Affiliated Hospital, Zhejiang University School of Medicine, Hangzhou, China; ^5^Department of Emergency Medicine, Zhongshan Hospital, Fudan University, Shanghai, China

**Keywords:** sepsis-induced cardiomyopathy, male, testosterone, sST2, mortality prediction, biomarkers

## Abstract

Sepsis-induced cardiomyopathy (SIC) is characterized by high mortality and poor outcomes. This study aimed to explore the relationship between testosterone and soluble ST2 (sST2) and all-cause mortality in patients with SIC. Clinical data from SIC patients at Renmin Hospital of Wuhan University from January 2021 and March 2023 were reviewed. Serum testosterone and sST2 were measured at admission. Kaplan–Meier analysis and receiver operative characteristic curve (ROC) were used to estimate the predictive values of testosterone and sST2 on 28 days and 90 days mortality of SIC. A total of 327 male subjects with SIC were enrolled in this study. During the 28 days and 90 days follow-up, 87 (26.6%) and 103 deaths (31.5%) occurred, respectively. Kaplan–Meier analysis showed significantly higher 28 days and 90 days survival in patients with higher testosterone and decreased sST2 levels (*p* < 0.001). Testosterone, sST2, and N-terminal pro-B-type natriuretic peptide (NT-proBNP) were significantly associated with 28 days and 90 days mortality (*p* < 0.05). Partial correlation analysis showed strong positive correlation between testosterone and left ventricular ejection fraction (LVEF) (*p* < 0.001), and negative correlation between testosterone and sST2 (*p* < 0.001), high-sensitivity troponin I (hs-TnI) levels (*p* < 0.001) and smoke history (*p* < 0.01). The concentrations of sST2 were positively related with *E*/*e*′ ratio (*p* < 0.001), and negatively correlated with TAPSE (*p* < 0.001). The combination of testosterone and sST2 enhanced the prediction of both 28 days [area under the ROC curve (AUC), 0.805] and 90 days mortality (AUC, 0.833). Early serum testosterone and sST2 levels could predict mortality of SIC independently and jointly. Further research is needed to determine the utility of biochemical markers in identifying high-risk patients with SIC.

## 1 Introduction

Sepsis remains the leading cause of mortality in intensive care units (ICUs) globally, resulting in 5 million deaths annually (1). Sepsis-induced cardiomyopathy (SIC) is a common and life-threatening complication of sepsis and manifests as left ventricular dysfunction, impaired ejection fraction and heart failure (2). The published incidence rate of SIC among septic patients is 28.0%–38.8% (3, 4). Despite the standardization of sepsis management, 70%–90% of mortality was reported in patients with SIC (5). Risk factors for developing SIC include elderly age, male sex, pre-existing heart failure, decreased systolic blood pressure, and elevated N-terminal pro-B-type natriuretic peptide (NT-proBNP) levels (6–8). The underlying mechanisms of SIC may involve myocardial suppression factors, abnormal calcium regulation, mitochondrial injury, and production of reactive oxygen species (9).

Studies showed that the incidence and mortality in male patients were higher than those in female patients (10, 11). Disruption of testosterone levels has been linked to worse prognosis in critically ill patients (11). Lazzerini et al. reported testosterone levels during inflammatory activation in men may increase the risk of Torsades de Pointes (TdP) by contributing to the overall prolonging effect of inflammation on QTc, possibly due to increasing conversion of testosterone to estrogen (12). The deficiency of testosterone was considered a risk factor for adverse cardiovascular events (13). However, data evaluating the prognostic predictive values of testosterone in patients with SIC are limited, and large-scale multicenter analysis is necessary.

sST2 is a novel biomarker for risk stratification, prognosis assessment, and therapeutic guidance in heart failure over the past decades (14). Previous data indicate that sST2 levels correlated with severity and mortality of sepsis, especially in patients with greater cardiovascular impairment (15). Compared with NT-proBNP and troponin T, sST2 levels may be a stronger independent predictor to evaluate the severity of inflammatory cardiomyopathy (16, 17). However, findings on sST2 in sepsis have been ambiguous. The study of Yang et al. reported that sST2 levels were not associated with cardiac function in septic patients (18). Studies determining the relationship between sST2 and SIC to predict prognosis in affected patients should be conducted.

Therefore, this prospective study aimed to measure the potential value of serum testosterone and sST2 concentrations for mortality prediction and risk evaluation of mortality in patients with SIC.

## 2 Methods

### 2.1 Study design

This prospective study enrolled 1,083 patients with suspected sepsis in the Department of Critical Care Medicine (*n* = 736), General ICU in East Campus (*n* = 128), Cardiovascular ICU (*n* = 96), Cardiovascular surgical ICU (*n* = 101), and Emergency ICU (*n* = 22) of Renmin Hospital of Wuhan University between January 2021 and March 2023. After excluding 756 patients, 327 male patients were diagnosed with SIC. The patients were followed up until 90 days after ICU admission. The investigators collected data, then blinded statisticians analyzed the data. The study protocol was approved by the Ethics Committee of Renmin Hospital of Wuhan University (Study No. WDRY2021-K094). This study was conducted following the International Conference on Harmonization Good Clinical Practice guideline. All patients or their authorized representatives provided written informed consent.

The inclusion criteria were (1) diagnosis of SIC based on a previous study (19): meeting the diagnostic definition of sepsis-3 (1); (2) LVEF <50%, or plasma hs-TnI > 0.78 ng/mL (normal range, 0–0.78 ng/mL) or NT-proBNP >500 pg/mL; (3) male sex; and (4) age between 18 and 80 years. The exclusion criteria were (1) previous diagnosis of cancer; (2) previously existing grade III-IV heart failure (New York Heart Association Classification); (3) dropped out during the 90 days follow-up, excluding death; (4) patients with missing data.

### 2.2 Clinical and laboratory data

Clinical information, including age, sex, body mass index (BMI), past medical history, including systolic (SBP) and diastolic blood pressure (DBP), were obtained from the electronic medical records. The values of white blood cells, serum creatinine, total bilirubin, NT-proBNP, hs-TnI, procalcitonin (PCT), C-reaction protein (CRP), serum amyloid A (SAA), and lactate were obtained from the laboratory reporting system. Furthermore, the scores of acute physiology and chronic health evaluation (APACHE) II and sequential organ failure assessment (SOFA), indicating the severity of illness and organ dysfunction/failure, were developed based on the above data.

### 2.3 Measurements of serum testosterone and sST2

After ICU admission, serum samples were immediately obtained and centrifuged, followed by storage at −80°C and thawed rapidly before test. Testosterone and sST2 levels in the baseline samples were measured using chemiluminescence (Siemens Atellica IM1600, Germany) and the immunofluorescence dry quantitative method i-CHROMATM (BodiTech, Guangxi, China), respectively.

### 2.4 Statistical analysis

Based on the distribution type of the measured variables, continuous variables were reported as mean and standard deviation (SD) or median and interquartile ranges (IQRs). Categorical variables were presented as frequencies and percentages. The Kolmogorov–Smirnov test was utilized to evaluate normally distributed values. Following the grouping method of Bidadkosh et al. (20), we grouped the whole population into T1 (lowest), T2 (middle) and T3 (highest) group based on tertiles of the values of testosterone and sST2. The chi-square test was utilized to compare dichotomous and categorical variables. The student’s *t*-test and Mann–Whitney *U*-test were employed to compare continuous data between groups, as appropriate. Moreover, the Kaplan–Meier method was used to calculate the survival time between groups. Univariate and multivariate Cox proportional hazards analysis was performed to evaluate the hazard ratio (HR) and 95% confidence intervals (CIs) for the association between testosterone or sST2 and mortality. To evaluate the relationship between testosterone and sST2 and clinical and echocardiographic parameters, Spearman’s rank correlation analyses were conducted with the “metadar” package in R statistical software. Receiver operating curve (ROC) analysis was performed to evaluate the sensitivity and specificity of testosterone and sST2 at different cut-off values, and the Youden index showed optimal cut-off values from the ROC for prognostic determination. A two-tailed *p* < 0.05 indicated statistical significance. SPSS software version 22.0, GraphPad Prism software version 9.0.0 and R version 4.2.3 statistical software were used for data filing and statistical analysis.

## 3 Results

### 3.1 Patients’ baseline characteristics

**Figure 1:**
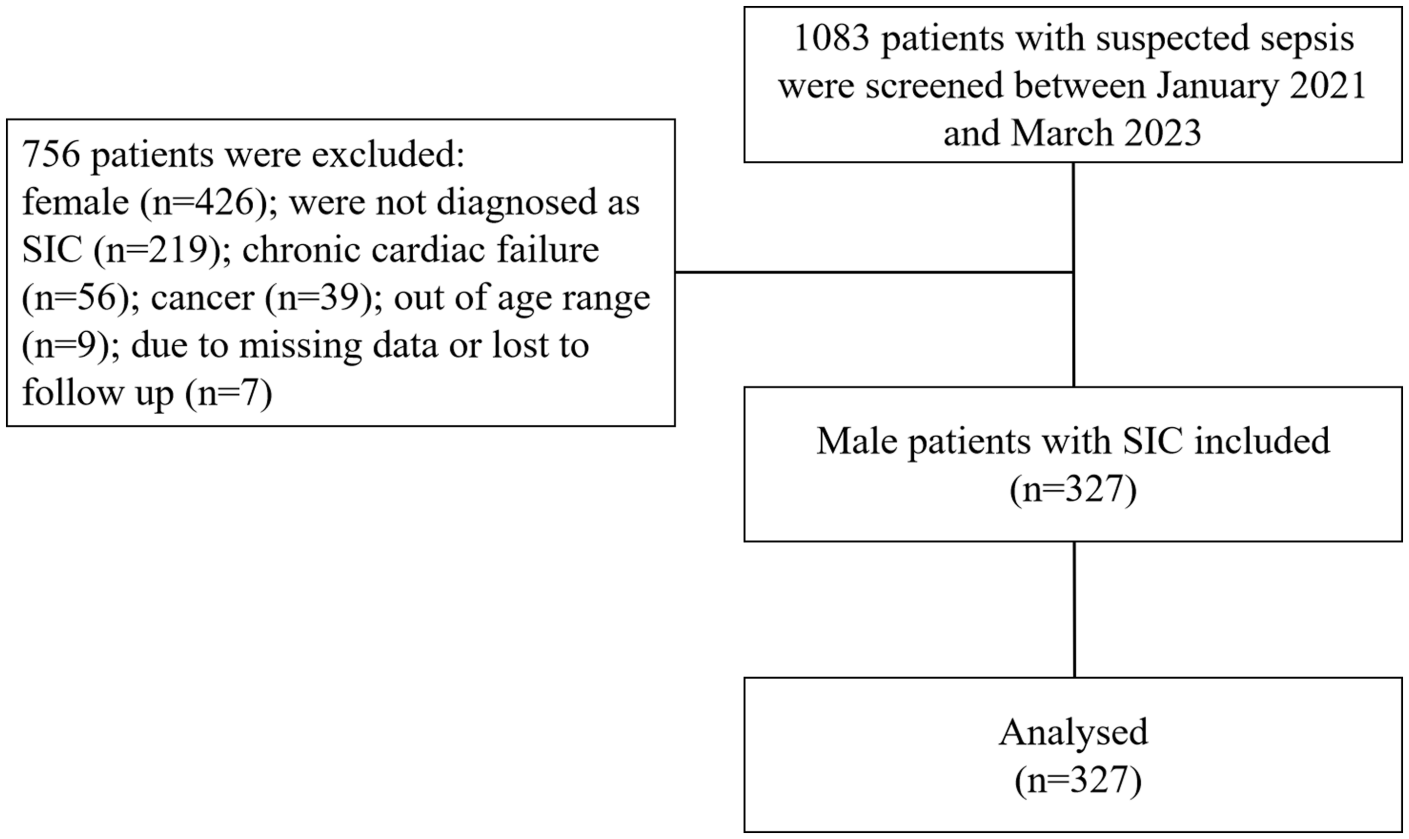
Flow diagram and outcome of the study participants. SIC, sepsis-induced cardiomyopathy.

The study flowchart are shown in Figure 1. The clinical and demographic characteristics of all study participants are listed in Table 1; Supplementary Table S1. A total of 327 male SIC patients were identified. The median age and BMI of the patients were 61 years and 21.7 kg/m^2^, respectively. Additionally, 98 patients were smokers (30.0%), and 56 patients had previous myocardial infarction (MI) history (17.1%). Furthermore, 26.6% (*n* = 87) and 31.5% (*n* = 103) of the patients died during the 28 days and 90 days follow-up (Table 1). The median serum testosterone and sST2 levels were 133.9 (IQR, 69.3–200.0) ng/dL and 153.2 (IQR, 63.0–405.8) ng/mL, respectively. The participants were grouped based on their serum testosterone and sST2 levels. Specifically, lower testosterone levels were related to a higher proportion of smoking habits, 28 days mortality, and 90 days mortality. Moreover, serum testosterone levels were correlated with SBP, DBP, LVEF, hs-TnI, PCT, CRP, SAA, lactate, and sST2, as well as APACHE II and SOFA scores (all *p* < 0.01). Meanwhile, sST2 concentration was also significantly associated with 28 days and 90 days mortality, SOFA score, NT-proBNP, hs-TnI, PCT, SAA, and lactate levels (all *p* < 0.05).

**Table 1:** Baseline characteristics of the study population.

Baseline characteristics	Overall (*n* = 327)	Testosterone tertiles, ng/dL			*p*-value	sST2 tertiles, ng/mL			*p*-value
		T1 (lowest)	T2 (middle)	T3 (highest)		T1 (lowest)	T2 (middle)	T3 (highest)	
Age (years)	61 (54, 68)	63 (53, 58)	62 (56, 69)	58 (53, 66)	0.193	59 (53, 67)	63 (56, 69)	61 (54, 68)	0.188
BMI (kg/m^2^)	21.7 (20.0, 23.4)	21.5 (20.0, 23.5)	22.3 (20.0, 23.7)	21.3 (19.8, 22.8)	0.092	22.0 (19.8, 23.2)	21.6 (20.0, 23.7)	21.7 (20.0, 23.5)	0.752
Smoke history	98 (30.0%)	45 (41.3%)	27 (24.8%)	26 (23.9%)	0.007^*^	30 (27.5%)	36 (33.0%)	32 (29.4%)	0.665
MI history	56 (17.1%)	17 (15.6%)	19 (17.4%)	20 (18.3%)	0.860	15 (13.8%)	25 (22.9%)	16 (14.7%)	0.141
28 days Mortality, n (%)	87 (26.6%)	53 (48.6%)	20 (18.3%)	14 (12.8%)	<0.001^*^	8 (7.3%)	20 (18.3%)	59 (54.1%)	<0.001^*^
90 days Mortality, n (%)	103 (31.5%)	64 (58.7%)	25 (22.9%)	14 (12.8%)	<0.001^*^	9 (8.3%)	24 (22.0%)	70 (64.2%)	<0.001^*^
Testosterone (ng/dL)	133.9 (69.3, 200.0)	50.8 (28.8, 70.0)	133.9 (108.5, 148.9)	238.9 (199.2, 262.4)	<0.001^*^	181.1 (121.8, 229.4)	117.3 (75.5, 187.6)	74.3 (39.2, 162.4)	<0.001^*^
sST2 (ng/mL)	153.2 (63.0, 405.8)	365.3 (177.3, 473.0)	94.0 (40.4, 294.2)	81.6 (43.8, 250.3)	<0.001^*^	41.8 (27.0, 64.1)	153.2 (102.2, 243.8)	470.3 (404.7, 699.3)	<0.001^*^
NT-proBNP (pg/mL)	3,845 (876,21,380)	4,113 (1,011,20,875)	3,954 (1,239,26,935)	2,599 (781,18,740)	0.231	2,323 (714,17,484)	5,246 (1,334,24,098)	3,535 (883,14,545)	0.045^*^
hs-TnI (ng/mL)	3.12 (0.45, 13.84)	9.47 (0.32, 26.17)	4.67 (0.16, 16.54)	1.92 (1.07, 4.17)	<0.001^*^	2.27 (1.15, 12.93)	1.59 (0.21, 9.13)	6.10 (1.09, 21.57)	0.004^*^
Lactate (mmol/L)	2.2 (1.7, 2.9)	2.6 (1.8, 3.7)	2.4 (1.9, 2.9)	1.8 (1.5, 2.4)	<0.001^*^	2.1 (1.7, 2.6)	2.2 (1.6, 2.7)	2.5 (1.8, 3.5)	0.001^*^
LVEF (%)	49 (44, 51)	46 (40, 51)	47 (41, 50)	50 (50, 51)	<0.001^*^	50 (45, 50)	49 (44, 53)	47 (42, 51)	0.280
LVESV (mL)	52.5 (36.5, 67.3)	55.2 (37.0, 68.1)	50.4 (36.0, 67.4)	52.2 (36.2, 67.0)	0.839	56.1 (38.5, 70.0)	35.2 (50.4, 66.5)	35.9 (53.2, 66.3)	0.332
*E*/*e*′ ratio	7.3 (5.2, 9)	7.4 (5.4, 9.1)	7.4 (5.4, 9.2)	7.0 (5.2, 8.9)	0.553	6.1 (4.2, 8.2)	7.3 (5.6, 9.0)	7.9 (5.9, 10.6)	<0.001^*^
TAPSE (mm)	20.3 (17.8, 23.7)	19.9 (17.8, 23.3)	21.5 (18.1, 23.7)	19.9 (17.6, 23.6)	0.514	22.5 (18.5, 24.0)	20.3 (17.5, 24.0)	19.5 (16.2, 22.6)	<0.001^*^

^*^Statistically significant at *p* < 0.05 level, two-sided. BMI, body mass index; MI, myocardial infarction; NT-proBNP, N-terminal pro B-type natriuretic peptide; hs-TnI, high-sensitivity troponin I; LVEF, left ventricular ejection fraction; LVESV, left ventricular end systolic volume; TAPSE, tricuspid annular pulmonary systolic excursion.

### 3.2 Survival analysis of testosterone and sST2 on mortality after 28 days and 90 days follow-ups

**Figure 2:**
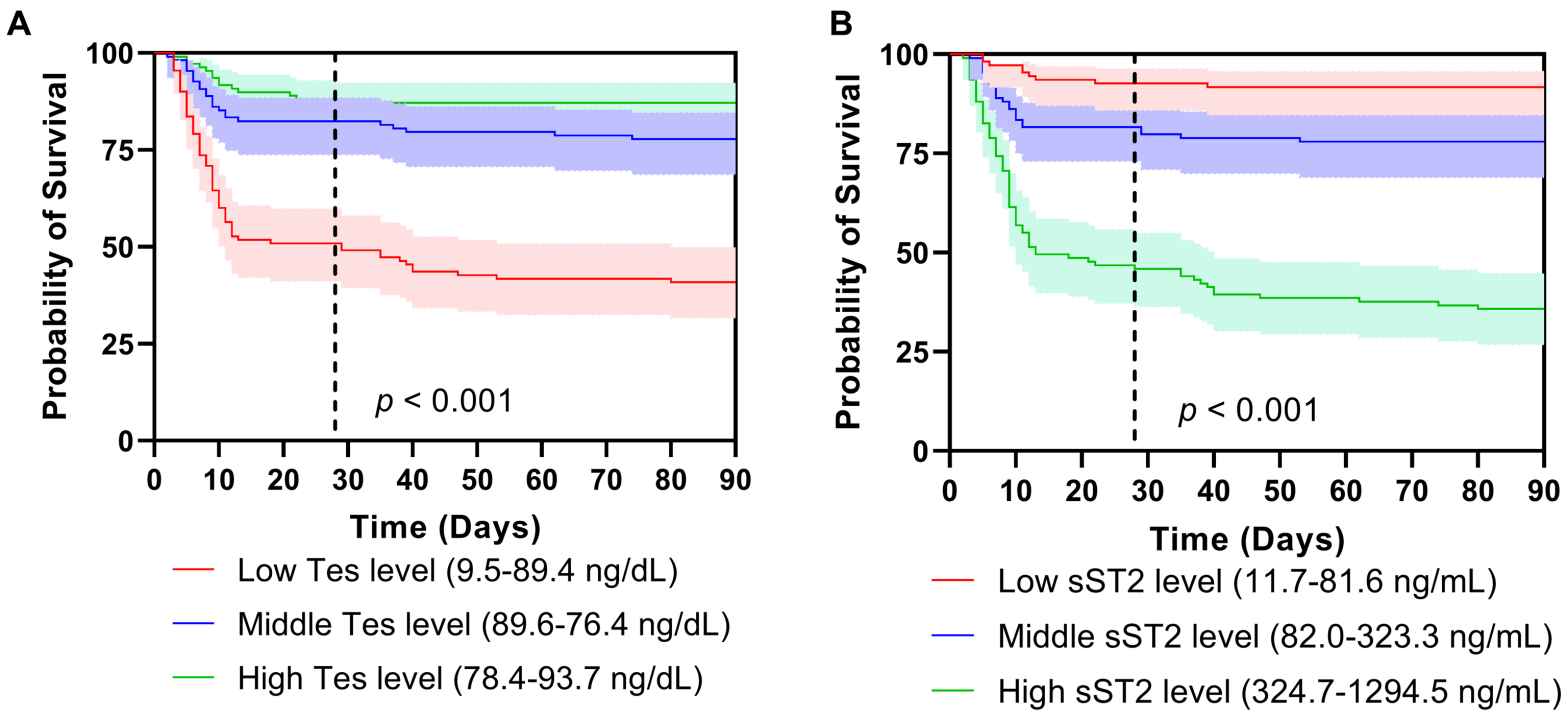
Survival curves of tertiles of testosterone **(A)** and sST2 **(B)** levels on mortality after 28 days **(A)** and 90 days follow-ups. Tes, testosterone.

The Kaplan–Meier analysis showed that the survival curves differed between groups. During the 28-day and 90-day follow-ups, patients with lower testosterone levels had significantly lower survival (*p* < 0.001, Figure 2A). The survival rate during the 28-day and 90-day follow-up was higher in the low tertiles of the sST2 group than in the middle and high tertiles of sST2 values (both *p* < 0.001, Figure 2B). Moreover, the univariate analyses showed that testosterone (HR, 0.994; 95% CI, 0.991–0.991, *p* < 0.001), sST2 (HR, 1.003; 95% CI, 1.002–1.004, *p* < 0.001), and NT-proBNP (HR, 1.000; 95% CI, 1.000–1.000; *p* < 0.001) levels were significantly associated with 28 days mortality (Table 2). In addition, BMI (HR, 1.084; 95% CI, 1.001–1.175, *p* = 0.048), testosterone (HR, 0.992; 95% CI, 0.989–0.995; *p* < 0.001), sST2 (HR 1.003; 95%CI, 1.002–1.003, *p* < 0.001), and NT-proBNP (HR, 1.000; 95%CI, 1.000–1.000, *p* < 0.001) were related with 90 days mortality (Table 2). The multivariate analysis showed that testosterone, sST2 and NT-proBNP levels were independently associated with 28-day and 90-day mortality (Table 2).

**Table 2:** Univariate and multivariate Cox regression model for 28 days and 90 days mortality in patients with SIC.

	Univariate analysis			Multivariate analysis		
Parameters	HR	95% CI	*p*-value	HR	95% CI	*p*-value
**28 days mortality risk**						
BMI	1.039	0.950–1.138	0.402			
Age	0.990	0.973–1.008	0.295			
Smoking habits	1.309	0.835–2.053	0.241			
Testosterone	0.994	0.991–0.991	<0.001^*^	0.993	0.990–0.996	<0.001^*^
sST2	1.003	1.002–1.004	<0.001^*^	1.003	1.002–1.003	<0.001^*^
NT-proBNP	1.000	1.000–1.000	<0.001^*^	1.000	1.000–1.000	<0.001^*^
hs-TnI	1.015	0.997–1.034	0.102			
**90 days mortality risk**						
BMI	1.084	1.001–1.175	0.048^*^			
Age	0.987	0.971–1.003	0.112			
Smoking habits	1.331	0.880–2.013	0.175			
Testosterone	0.992	0.989–0.995	<0.001^*^	0.991	0.989–0.994	<0.001^*^
sST2	1.003	1.002–1.003	<0.001^*^	1.003	1.002–1.003	<0.001^*^
NT-proBNP	1.000	1.000–1.000	<0.001^*^	1.000	1.000–1.000	<0.001^*^
hs-TnI	1.013	0.996–1.030	0.132			

^*^Statistically significant at *p* < 0.05 level, two-sided.

### 3.3 Associations of testosterone and sST2 and clinical and echocardiographic parameters in SIC patients

**Figure 3:**
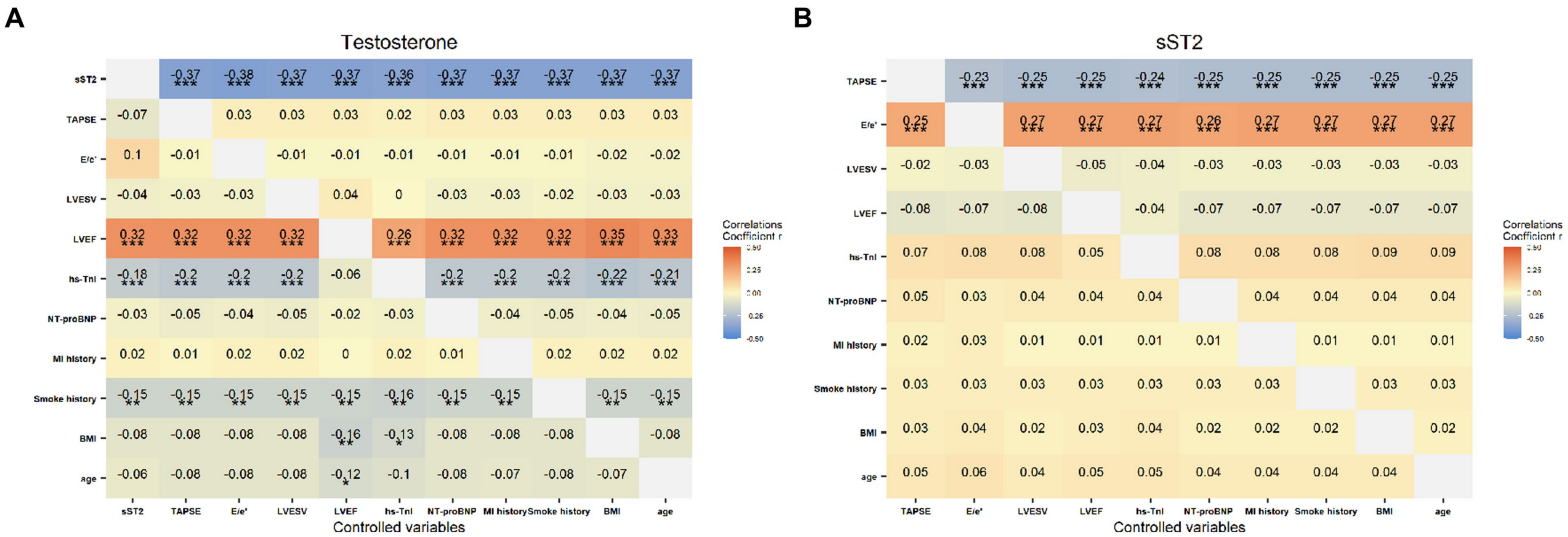
The correlation of the testosterone (A) and sST2 (B) levels with clinical characteristics (MI history, smoke history, BMI, age), laboratory (hs-TnI, NT-proBNP) and echocardiographic indicators (TAPSE, E/e’ ratio, LVEWV, LVEF), respectively. Color depth correspond to the strength of the correlation coefficients. ^*^p < 0.05, ^**^p < 0.01 and ^***^p < 0.001.

Results from the covariance analysis showing partial correlation coefficients between testosterone and sST2 and the clinical and echocardiographic parameters are presented in Figure 3. A strong positive correlation was found between testosterone and LVEF (*p* < 0.001), and a negative correlation between testosterone and sST2 (*p* < 0.001), hs-TnI levels (*p* < 0.001) and smoke history (*p* < 0.01). Meanwhile, the sST2 concentrations were positively related with *E*/*e*′ ratio (*p* < 0.001), and negatively correlated with TAPSE (*p* < 0.001).

### 3.4 Receiver operating characteristic curve analysis of 28 days and 90 days mortality in SIC

**Figure 4:**
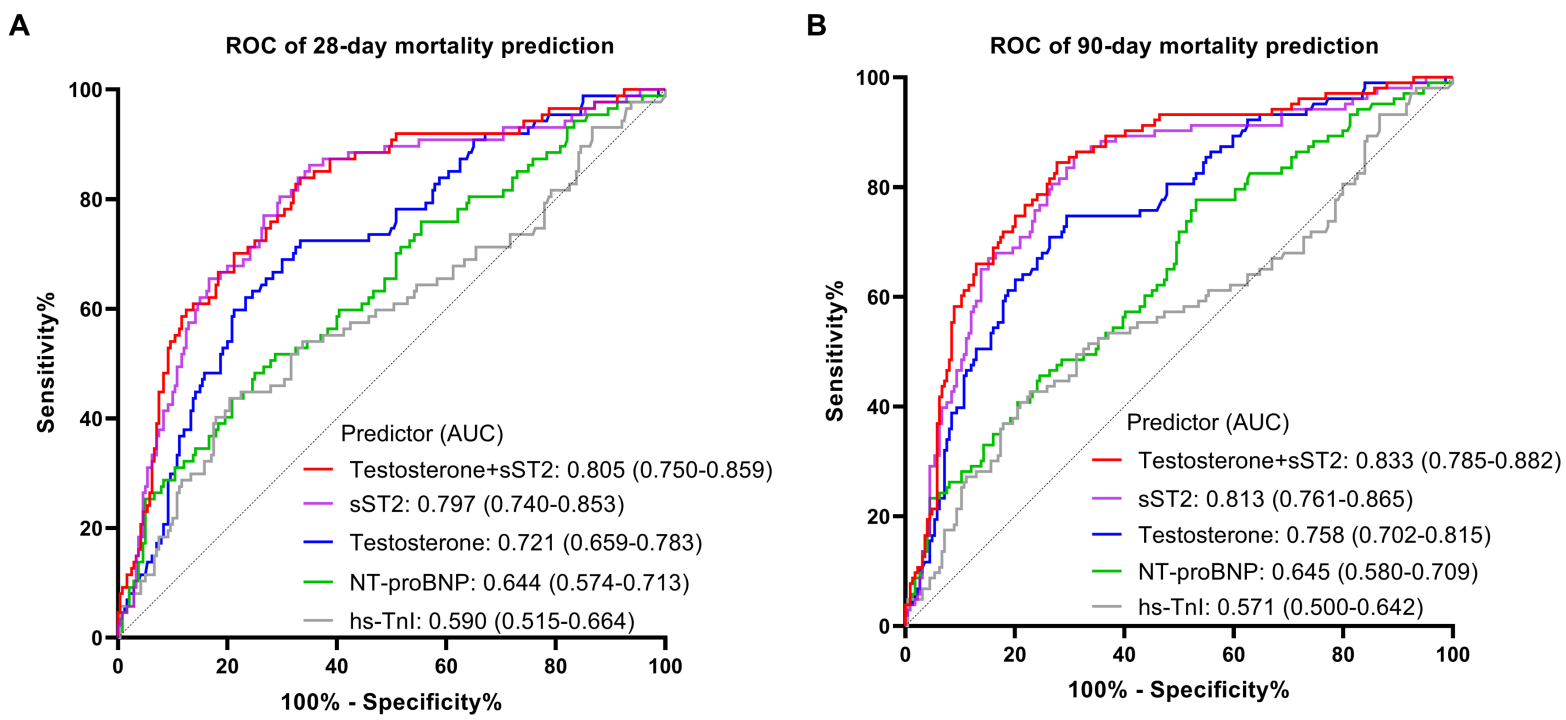
The ROC curve analysis of sST2, testosterone, hs-TnI, NT-proBNP for the prognostic value of 28-day mortality **(A)** and 90-day mortality **(B)** in patients with SIC. ROC, receiver operating characteristic; AUC, area under the curve; SIC, sepsis induced cardiomyopathy.

The ROC curves were plotted, and the AUCs were analyzed to further identify the mortality-predictive abilities of the two biomarkers. The AUCs of testosterone and sST2 were higher than NT-proBNP and hs-TnI for predicting 28 days and 90 days mortality in SIC (Figure 4). Moreover, the AUC of the combination of testosterone and sST2 for predicting 28 days and 90 days mortality were 0.805 (95% CI, 0.750–0.859) and 0.833 (95% CI, 0.785–0.882), respectively (Figure 4). The optimal cut-off values of testosterone and sST2 for predicting 28 days and 90 days mortality were 112.4 ng/dL (28 days mortality, AUC = 0.721; 90 days mortality, AUC = 0.758) and 168.9 ng/mL (28 days mortality, AUC = 0.797; 90 days mortality, AUC = 0.813), respectively (Figure 4 and Table 3).

**Table 3:** The ROC curve analysis of sST2, testosterone, NT-proBNP, hs-TnI for the prognostic value of 28 days mortality and 90 days mortality in patients with SIC.

Parameters	AUC (95% CI)	Optimal cut-off	Sensitivity (%)	Specificity (%)	*p*-value
**28 days mortality prediction**					
sST2 (ng/mL)	0.797 (0.740–0.853)	>168.9	86.21	65.00	<0.001^*^
Testosterone (ng/dL)	0.721 (0.659–0.783)	<112.4	72.41	66.67	<0.001^*^
NT-proBNP (pg/mL)	0.644 (0.574–0.713)	>8,763	48.28	75.00	<0.001^*^
hs-TnI (ng/mL)	0.590 (0.515–0.664)	>13.3	43.68	79.58	0.013^*^
**90 days mortality prediction**					
sST2 (ng/mL)	0.813 (0.761–0.865)	>168.9	86.41	68.75	<0.001^*^
Testosterone (ng/dL)	0.758 (0.702–0.815)	<112.4	74.76	70.54	<0.001^*^
NT-proBNP (pg/mL)	0.571 (0.500–0.642)	>2,185	77.67	46.88	<0.001^*^
hs-TnI (ng/mL)	0.645 (0.580–0.709)	>11.27	42.72	77.23	0.039^*^

^*^Statistically significant at *p* < 0.05 level, two-sided. ROC, receiver operating characteristic; NT-proBNP, N-terminal pro B-type natriuretic peptide; hs-TnI, high-sensitivity troponin I; SIC, sepsis induced cardiomyopathy; AUC, area under the curve; CI, confidence interval.

## 4 Discussion

SIC, a frequent sepsis complication, results in left heart failure with decreased pumping ability and right heart failure with fluid overload. SIC is strongly associated with end-organ hypoperfusion and heart damage from inflammation (3). Despite receiving fluid resuscitation and standard care, SIC leads to poor outcomes and high mortality. Thus, exploring the potential risk biomarkers of male SIC patients is crucial. Endocrine dysfunction is related to the mortality and clinical outcomes in critically ill patients (21). Male patients showed higher mortality risk due to altered testosterone levels (10). The current analysis showed that decreased testosterone levels were related to severity and mortality in critically ill male patients with SIC. Furthermore, testosterone levels were associated with traditional cardiac biomarkers and echocardiography parameters in patients with SIC. To the best of our knowledge, this study contributes to the existing data by associating testosterone with clinical prognosis prediction among patients with SIC.

Previous research has demonstrated that testosterone plays a critical role in the preservation of cardiovascular homeostasis during inflammation. Castration-induced testosterone suppression in lipopolysaccharide-treated rats could lower blood pressure, raise neuronal inflammatory markers, and time- and frequency-domain measures of heart rate variability (22). On the other hand, testosterone supplementation mitigated superoxide-provoked apoptotic and necrotic cell death by upregulating the pro-survival Akt/NF-κB pathway and decreasing the pro-apoptotic enzyme caspase-3 in cardiac myocytes (23). In male patients with gastrointestinal infections, testosterone mitigates Th2-dominant responses and promotes Th1 immunity by modulating androgen receptor signaling and cytokine secretion in macrophages and lymphocytes (24). Temporarily suppressing inflammation leads to early hormone resistance, which makes testosterone deficiency a potential prognostic indicator of progressive sepsis and SIC.

Lower testosterone levels between 9.5 and 89.4 ng/dL were linked to greater inflammation and worse outcomes in septic cardiomyopathy, similar to coronavirus disease 2019 (COVID-19) (25). Earlier research has shown that decreased testosterone levels were associated with increased CRP, PCT, and interleukin (IL)-6 levels in patients with COVID-19 (25, 26). Furthermore, lower testosterone levels correlated with higher APACHE II and SOFA scores and independently predicted higher mortality in patients with SIC (27). Thus, declining testosterone levels could indicate worsening sepsis severity and increased mortality risk in male patients.

Moreover, conventional cardiac biomarkers in mortality prediction of SIC are not satisfactory (28). Chen et al. (29) reported the AUC for BNP and cTnI in SIC 28 days mortality predicting were 0.571, and 0.544, respectively. The current analysis found that the AUC for testosterone predicting 28 days mortality of SIC was 0.721, which was better than NT-proBNP (0.644) and hs-TnI (0.590). Thus, testosterone may have superior prognostic value compared with these established cardiac biomarkers for predicting SIC mortality.

Our analysis also found that sST2 level could serve as a potentially useful biomarker to predict outcomes in patients with SIC. sST2 inhibits immune responses and results in myocardial injury by targeting ST2L/IL-33 interaction (14). Although sST2 is an inflammation biomarker in other conditions (16, 17, 30, 31), its role in SIC is unclear. A previous study found sST2 elevation on days 1 and 3 of septic patients and correlated with lactate,which indicating illness severity (15). In patients with severe COVID-19, sST2 was also found to be upregulated and positively correlated with inflammatory markers (CRP and PCT), while negatively related to T lymphocyte counts (31, 32). Our results indicated that higher sST2 levels in patients with SIC positively correlate with *E*/*e*′ ratio and negatively correlate with TAPSE, suggesting that sST2 may be a potential biomarker reflecting cardiac injury in sepsis. ROC analysis has indicated that sST2 has a higher predictive value for mortality than traditional biomarkers. Thus, sST2 could enhance clinical predictive algorithms under heterogeneous sepsis conditions. Moreover, the combination of testosterone and sST2 improves mortality prediction in SIC patients.

However, our study has several limitations. Firstly, due to the lack of a universally accepted definition of SIC, the inclusion criteria for the current project referenced a study from Mayo Clinic (19), which may require further investigation to accurately identify the population affected by SIC. Secondly, biomarker values were measured at a single time point, limiting insights into the dynamic immune response in SIC over time. Moreover, altered biomarker levels may represent compensatory mechanisms early in SIC rather than a pathological effect. However,the cross-sectional design cannot prove a causal relationship between testosterone, sST2, and the outcomes. In summary, larger prospective multicenter studies measuring serial biomarker levels and long-term outcomes are warranted to validate these biomarkers as predictive tools.

## 5 Conclusion

Decreased testosterone and elevated sST2 may predict prognosis in patients with SIC. These biomarkers could enable risk stratification and potentially guide treatment, leading to new immunomodulatory therapies. However, further studies are necessary to confirm their diagnostic and prognostic values before clinical implementation.

## Data availability statement

The original contributions presented in the study are included in the article/Supplementary material, further inquiries can be directed to the corresponding authors.

## Ethics statement

The studies involving humans were approved by the Ethics Committee of Renmin Hospital of Wuhan University. The studies were conducted in accordance with the local legislation and institutional requirements. The participants provided their written informed consent to participate in this study.

## Author contributions

LW: Conceptualization, Formal analysis, Project administration, Resources, Writing – original draft. WD: Formal analysis, Writing – review & editing. RZ: Resources, Writing – original draft. TL: Formal analysis, Writing – review & editing. ZZ: Project administration, Writing – review & editing. ZS: Project administration, Writing – review & editing. SM: Project administration, Writing – review & editing. SW: Data curation, Investigation, Writing – review & editing. HW: Project administration, Formal analysis, Writing – review & editing. JL: Resources, Formal analysis, Writing – review & editing. JZ: Resources, Project administration, Writing – review & editing. WX: Data curation, Resources, Writing – review & editing. GL: Data curation, Resources, Writing – review & editing. WG: Data curation, Resources, Writing – review & editing. HZ: Resources, Data curation, Writing – review & editing. YL: Formal analysis, Project administration, Writing – review & editing. LZ: Formal analysis, Investigation, Project administration, Resources, Supervision, Writing – review & editing.

## References

[1] Seymour CW Liu VX Iwashyna TJ Brunkhorst FM Rea TD Scherag A. Assessment of clinical criteria for sepsis: for the third international consensus definitions for sepsis and septic shock (sepsis-3). JAMA. (2016) 315:762–74. doi: 10.1001/jama.2016.0288, PMID: 26903335 PMC5433435

[2] Markwart R Saito H Harder T Tomczyk S Cassini A Fleischmann-Struzek C. Epidemiology and burden of sepsis acquired in hospitals and intensive care units: a systematic review and meta-analysis. Intensive Care Med. (2020) 46:1536–51. doi: 10.1007/s00134-020-06092-1, PMID: 32591853 PMC7381455

[3] Lin YM Lee MC Toh HS Chang WT Chen SY Kuo FH. Association of sepsis-induced cardiomyopathy and mortality: a systematic review and meta-analysis. Ann Intensive Care. (2022) 12:112. doi: 10.1186/s13613-022-01089-3, PMID: 36513882 PMC9748009

[4] Vieillard-Baron A Caille V Charron C Belliard G Page B Jardin F. Actual incidence of global left ventricular hypokinesia in adult septic shock. Crit Care Med. (2008) 36:1701–6. doi: 10.1097/CCM.0b013e318174db05, PMID: 18496368

[5] L’Heureux M Sternberg M Brath L Turlington J Kashiouris MG. Sepsis-induced cardiomyopathy: a comprehensive review. Curr Cardiol Rep. (2020) 22:35. doi: 10.1007/s11886-020-01276-2, PMID: 32377972 PMC7222131

[6] Liang YW Zhu YF Zhang R Zhang M Ye XL Wei JR. Incidence, prognosis, and risk factors of sepsis-induced cardiomyopathy. World J Clin Cases. (2021) 9:9452–68. doi: 10.12998/wjcc.v9.i31.9452, PMID: 34877280 PMC8610866

[7] Jeong HS Lee TH Bang CH Kim JH Hong SJ. Risk factors and outcomes of sepsis-induced myocardial dysfunction and stress-induced cardiomyopathy in sepsis or septic shock: a comparative retrospective study. Medicine. (2018) 97:e0263. doi: 10.1097/MD.0000000000010263, PMID: 29595686 PMC5895365

[8] Song MJ Lee SH Leem AY Kim SY Chung KS Kim EY. Predictors and outcomes of sepsis-induced cardiomyopathy in critically ill patients. Acute Crit Care. (2020) 35:67–76. doi: 10.4266/acc.2020.00024, PMID: 32407613 PMC7280797

[9] Hollenberg SM Singer M. Pathophysiology of sepsis-induced cardiomyopathy. Nat Rev Cardiol. (2021) 18:424–34. doi: 10.1038/s41569-021-00541-4 33473203

[10] Vázquez-Martínez ER García-Gómez E Camacho-Arroyo I González-Pedrajo B. Sexual dimorphism in bacterial infections. Biol Sex Differ. (2018) 9:27. doi: 10.1186/s13293-018-0186-9, PMID: 29925409 PMC6011518

[11] Chicco D Jurman G. Survival prediction of patients with sepsis from age, sex, and septic episode number alone. Sci Rep. (2020) 10:17156. doi: 10.1038/s41598-020-74068-9, PMID: 33051513 PMC7555553

[12] Lazzerini PE Cantara S Bertolozzi I Accioli R Salvini V Cartocci A. Transient hypogonadism is associated with heart rate-corrected QT prolongation and Torsades de Pointes risk during active systemic inflammation in men. J Am Heart Assoc. (2022) 11:e023371. doi: 10.1161/JAHA.121.023371, PMID: 34935398 PMC9075210

[13] Boden WE Miller MG McBride R Harvey C Snabes MC Schmidt J. Testosterone concentrations and risk of cardiovascular events in androgen-deficient men with atherosclerotic cardiovascular disease. Am Heart J. (2020) 224:65–76. doi: 10.1016/j.ahj.2020.06.007, PMID: 32335402

[14] Lotierzo M Dupuy AM Kalmanovich E Roubille F Cristol JP. sST2 as a value-added biomarker in heart failure. Clin Chim Acta. (2020) 501:120–30. doi: 10.1016/j.cca.2019.10.029, PMID: 31678574

[15] Hur M Kim H Kim HJ Yang HS Magrini L Marino R. Soluble ST2 has a prognostic role in patients with suspected sepsis. Ann Lab Med. (2015) 35:570–7. doi: 10.3343/alm.2015.35.6.570, PMID: 26354344 PMC4579100

[16] Obradovic DM Büttner P Rommel KP Blazek S Loncar G von Haehling S. Soluble ST2 receptor: biomarker of left ventricular impairment and functional status in patients with inflammatory cardiomyopathy. Cells. (2022) 11:414. doi: 10.3390/cells11030414, PMID: 35159224 PMC8833891

[17] Coronado MJ Bruno KA Blauwet LA Tschöpe C Cunningham MW Pankuweit S. Elevated sera sST2 is associated with heart failure in men ≤50 years old with myocarditis. J Am Heart Assoc. (2019) 8:e008968. doi: 10.1161/JAHA.118.008968, PMID: 30638108 PMC6497352

[18] Yang HS Hur M Kim H Magrini L Marino R Di Somma S. Soluble suppression of tumorigenicity 2 and echocardiography in sepsis. Ann Lab Med. (2016) 36:590–4. doi: 10.3343/alm.2016.36.6.590, PMID: 27578513 PMC5011113

[19] Wang L Xie W Li G Hu B Wu W Zhan L. Lipocalin 10 as a new prognostic biomarker in sepsis-induced myocardial dysfunction and mortality: a pilot study. Mediat Inflamm. (2021) 2021:6616270. doi: 10.1155/2021/6616270 PMC8166480 34121925

[20] Bidadkosh A Lambooy SPH Heerspink HJ Pena MJ Henning RH Buikema H. Predictive properties of biomarkers GDF-15, NTproBNP, and hs-TnT for morbidity and mortality in patients with type 2 diabetes with nephropathy. Diabetes Care. (2017) 40:784–92. doi: 10.2337/dc16-2175 28341782

[21] Zhang MQ Macala KF Fox-Robichaud A Mendelson AA Lalu MM. Sepsis Canada National Preclinical Sepsis Platform. Sex- and gender-dependent differences in clinical and preclinical sepsis. Shock. (2021) 56:298–307. doi: 10.1097/SHK.0000000000001708 33399356

[22] Sallam MY El-Gowilly SM El-Mas MM. Cardiac and brainstem neuroinflammatory pathways account for androgenic incitement of cardiovascular and autonomic manifestations in endotoxic male rats. J Cardiovasc Pharmacol. (2021) 77:632–41. doi: 10.1097/FJC.0000000000000993 33852527

[23] Xiao FY Nheu L Komesaroff P Ling S. Testosterone protects cardiac myocytes from superoxide injury via NF-κB signalling pathways. Life Sci. (2015) 133:45–52. doi: 10.1016/j.lfs.2015.05.009, PMID: 26032259

[24] Takahashi T Iwasaki A. Sex differences in immune responses. Science. (2021) 371:347–8. doi: 10.1126/science.abe7199 33479140

[25] Salciccia S Del Giudice F Eisenberg ML Mastroianni CM De Berardinis E Ricciuti GP. Testosterone target therapy: focus on immune response, controversies and clinical implications in patients with COVID-19 infection. Ther Adv Endocrinol Metab. (2021) 12:20420188211010105. doi: 10.1177/20420188211010105, PMID: 34104394 PMC8072920

[26] Vena W Pizzocaro A Maida G Amer M Voza A Di Pasquale A. Low testosterone predicts hypoxemic respiratory insufficiency and mortality in patients with COVID-19 disease: another piece in the COVID puzzle. J Endocrinol Investig. (2022) 45:753–62. doi: 10.1007/s40618-021-01700-7, PMID: 34792796 PMC8600346

[27] Birtolo MF Vena W Pizzocaro A Lavezzi E Brunetti A Jaafar S. Serum testosterone mirrors inflammation parameters in females hospitalized with COVID-19. J Endocrinol Investig. (2023) 46:939–45. doi: 10.1007/s40618-022-01957-6, PMID: 36370325 PMC9660177

[28] Méndez Hernández R Ramasco RF. Biomarkers as prognostic predictors and therapeutic guide in critically ill patients: clinical evidence. J Pers Med. (2023) 13:333. doi: 10.3390/jpm13020333, PMID: 36836567 PMC9965041

[29] Chen FC Xu YC Zhang ZC. Multi-biomarker strategy for prediction of myocardial dysfunction and mortality in sepsis. J Zhejiang Univ Sci B. (2020) 21:537–48. doi: 10.1631/jzus.B2000049, PMID: 32633108 PMC7383321

[30] Van Nynatten LR Slessarev M Martin CM Leligdowicz A Miller MR Patel MA. Novel plasma protein biomarkers from critically ill sepsis patients. Clin Proteomics. (2022) 19:50. doi: 10.1186/s12014-022-09389-3, PMID: 36572854 PMC9792322

[31] Hoogerwerf JJ Tanck MW van Zoelen MA Wittebole X Laterre PF van der Poll T. Soluble ST2 plasma concentrations predict mortality in severe sepsis. Intensive Care Med. (2010) 36:630–7. doi: 10.1007/s00134-010-1773-0, PMID: 20151106 PMC2837188

[32] Zeng Z Hong XY Li Y Chen W Ye G Li Y. Serum-soluble ST2 as a novel biomarker reflecting inflammatory status and illness severity in patients with COVID-19. Biomark Med. (2020) 14:1619–29. doi: 10.2217/bmm-2020-0410, PMID: 33336592

