# Supplementary material for: Testosterone and soluble ST2 as mortality predictive biomarkers in male patients with sepsis-induced cardiomyopathy
Source: Front Med (Lausanne). 2024 Jan 8;10:1278879. doi: 10.3389/fmed.2023.1278879 (PMC10801257; doi:10.3389/fmed.2023.1278879)
Supplement: Supplementary file 1 [file Table_1.docx]

Supplementary Material

# Supplementary Tables

Supplementary Table 1 .Baseline characteristics of the study population.

| **Baseline characteristics** | **Overall (n=327)** | **Testosterone tertiles, ng/dL** | | | ***p* value** | **sST2 tertiles, ng/mL** | | | ***p* value** |
| --- | --- | --- | --- | --- | --- | --- | --- | --- | --- |
|  |  | **T1 (lowest)** | **T2 (middle)** | **T3 (highest)** |  | **T1 (lowest)** | **T2 (middle)** | **T3 (highest)** |  |
| Hospital length of stay (days) | 12 (9,16) | 12 (9,15) | 12 (9,16) | 12 (9,16) | 0.871 | 12 (9,16) | 13 (9,16) | 11 (9,14) | 0.093 |
| ICU length of stay (days) | 6 (5,9) | 5 (4,9) | 6 (4,10) | 6 (4,9) | 0.276 | 6 (4,9) | 6 (4,10) | 6 (4,9) | 0.670 |
| HR (bpm) | 94 (80,112) | 97 (83,116) | 93 (82,112) | 90 (79,106) | 0.166 | 92 (80,110) | 95 (83,112) | 92 (80,113) | 0.759 |
| CVP (cmH_2_O) | 7 (5,10) | 6 (4,9) | 7 (5,10) | 7 (5,11) | 0.011* | 8 (5,11) | 7 (5,10) | 7 (4,9) | 0.140 |
| SBP (mmHg) | 112±19 | 106±22 | 115±16 | 114±15 | <0.001* | 116±16 | 109±17 | 111±21 | 0.016* |
| DBP (mmHg) | 61±15 | 55±14 | 65±15 | 63±13 | <0.001* | 63±14 | 58±14 | 62±16 | 0.092 |
| APACHEⅡscore | 16 (13,21) | 18 (14,23) | 15 (12,20) | 16 (13,21) | 0.005* | 18 (12,21) | 16 (13,22) | 16 (13,21) | 0.937 |
| SOFA score | 7 (5,10) | 9 (6,11) | 6 (5,9) | 7 (6,10) | <0.001* | 7 (5,9) | 7 (5,10) | 8 (6.10) | 0.038* |
| WBC (10^9^/L) | 15.3 (9.7,20.1) | 14.2 (8.8,19.4) | 15.4 (9.8,21.0) | 15.3 (11.0,19.6) | 0.341 | 17.0 (10.3,20.8) | 15.1 (10.2,18.3) | 14.2 (8.4,19.3) | 0.124 |
| sCr (μmol/L) | 106.0 (77.0,134.0) | 104.0 (72.5,137.0) | 114.0 (84.5,132.0) | 104.0 (70.5,133.5) | 0.333 | 107.0 (83.5,139.0) | 103.0 (76.0,132.0) | 106.0 (69.5,133.5) | 0.240 |
| Total bilirubin (mg/dl) | 1.05 (0.64,1.46) | 1.05 (0.64,1.43) | 1.05 (0.70,1.49) | 1.05 (0.64,1.55) | 0.779 | 0.99 (0.64,1.52) | 1.11 (0.64,1.49) | 1.05 (0.70, 1.40) | 0.925 |
| PCT (ng/mL) | 4.45 (2.63,10.22) | 10.01 (5.58,12.64) | 4.45 (3.02,10.39) | 2.66 (1.51,3.97) | <0.001* | 3.66 (2.14,5.95) | 4.82 (2.65,10.18) | 5.61 (3.12,11.68) | 0.003* |
| CRP (mg/L) | 90.7 (48.8,236.6) | 215.2 (81.9,317.3) | 108.9 (58.3,218.1) | 53.5 (21.7,86.5) | <0.001* | 82.99 (43.86,177.30) | 96.1 (55.8,242.3) | 94.8 (44.3,271.1) | 0.099 |
| SAA (mg/L) | 250.7 (145.6,450.9) | 312.0 (216.9,582.7) | 340.6 (166.5,495,4) | 174.1 (89.6,246.7) | <0.001* | 222.8 (116.6,341.6) | 247.2 (129.2,440.5) | 325.4 (200.7,563.9) | <0.001* |

* Statistically significant at *p* < 0.05 level, two-sided.

ICU, intensive care unit;CVP, [central venous pressure](https://www.bing.com/ck/a?!&&p=86dbdb0dcca1e216JmltdHM9MTY4NDk3MjgwMCZpZ3VpZD0xZmRjOGZjZi0xN2FlLTY4OWEtMWI5My05ZDNiMTZlNDY5NWYmaW5zaWQ9NTI1NA&ptn=3&hsh=3&fclid=1fdc8fcf-17ae-689a-1b93-9d3b16e4695f&psq=CVP&u=a1aHR0cHM6Ly9udXJzZWtleS5jb20vY2VudHJhbC12ZW5vdXMtcHJlc3N1cmUtbW9uaXRvcmluZy8&ntb=1); SBP, systolic blood pressure; DBP, diastolic blood pressure; APACHEⅡscore, acute physiology and chronic health evaluation II score; SOFA score, sequential organ failure assessment score; WBC, white blood cells; sCr, serum creatinine; PCT, procalcitonin; CRP, C-reaction protein; SAA, serum amyloid A.
